# Comprehensive Profiling of Secreted Factors in the Cerebrospinal Fluid of Moyamoya Disease Patients

**DOI:** 10.1007/s12975-023-01135-7

**Published:** 2023-02-06

**Authors:** Kumar Abhinav, Alex G. Lee, Arjun V. Pendharkar, Mark Bigder, Anthony Bet, Yael Rosenberg-Hasson, Michelle Y. Cheng, Gary K. Steinberg

**Affiliations:** 1 Department of Neurosurgery, Stanford University School of Medicine, 1201 Welch Road, MSLS P305, Stanford, CA 94305 USA; 2 Stanford Stroke Center, Stanford University School of Medicine, Stanford, CA USA; 3 Present Address: Department of Neurosurgery, Bristol Institute of Clinical Neuroscience, Southmead Hospital, Bristol, UK; 4 Division of Hematology and Oncology, Department of Pediatrics, University of California, San Francisco, CA USA; 5 Human Immune Monitoring Center, Stanford University School of Medicine, Stanford, CA USA

**Keywords:** Moyamoya disease, Neuroinflammation, Revascularization, Cerebrospinal fluid, Stroke, Surgical outcome

## Abstract

Moyamoya disease (MMD) is characterized by progressive occlusion of the intracranial internal carotid arteries, leading to ischemic and hemorrhagic events. Significant clinical differences exist between ischemic and hemorrhagic MMD. To understand the molecular profiles in the cerebrospinal fluid (CSF) of MMD patients, we investigated 62 secreted factors in both MMD subtypes (ischemic and hemorrhagic) and examined their relationship with preoperative perfusion status, the extent of postoperative angiographic revascularization, and functional outcomes. Intraoperative CSF was collected from 32 control and 71 MMD patients (37 ischemic and 34 hemorrhagic). Multiplex Luminex assay analysis showed that 41 molecules were significantly elevated in both MMD subtypes when compared to controls, including platelet-derived growth factor-BB (PDGF-BB), plasminogen activator inhibitor 1 (PAI-1), and intercellular adhesion molecule 1 (ICAM1) (*p* < 0.001). Many of these secreted proteins have not been previously reported in MMD, including interleukins (IL-2, IL-4, IL-5, IL-7, IL-8, IL-9, IL-17, IL-18, IL-22, and IL-23) and C-X-C motif chemokines (CXCL1 and CXCL9). Pathway analysis indicated that both MMD subtypes exhibited similar cellular/molecular functions and pathways, including cellular activation, migration, and inflammatory response. While neuroinflammation and dendritic cell pathways were activated in MMD patients, lipid signaling pathways involving nuclear receptors, peroxisome proliferator-activated receptor (PPAR), and liver X receptors (LXR)/retinoid X receptors (RXR) signaling were inhibited. IL-13 and IL-2 were negatively correlated with preoperative cerebral perfusion status, while 7 factors were positively correlated with the extent of postoperative revascularization. These elevated cytokines, chemokines, and growth factors in CSF may contribute to the pathogenesis of MMD and represent potential future therapeutic targets.

**Supplementary Information:**

The online version contains supplementary material available at 10.1007/s12975-023-01135-7.

## Introduction

MMD is a chronic cerebrovascular disease characterized by progressive occlusion of the intracranial internal carotid arteries, leading to ischemic and hemorrhagic strokes. The resulting progressive hypoperfusion results in the formation of multiple collateral vessels at the base of the brain, leading to the characteristic “puff of smoke” angiographic appearance [1, 2]. The disease can present with ischemic or hemorrhagic symptoms. The only effective treatment modality to prevent future vascular events is surgical revascularization. The resulting blood flow augmentation using extracranial to intracranial (EC-IC) bypass procedures can be achieved using direct or indirect revascularization or a combination of the two techniques [3]. Direct bypass procedure has been demonstrated to be superior in reducing hemorrhagic events in hemorrhagic MMD compared to conservative management [4, 5]. In ischemic MMD, our own experience favors the use of direct bypass to provide a greater degree of revascularization with no increase in perioperative stroke risk in comparison with indirect bypass [3].

The pathophysiology of MMD is largely unclear. Approximately 10–15% of MMD patients exhibit a familial occurrence [6, 7]. Genetic studies have revealed several genes associated with MMD [6, 7]. *Ring finger protein 213 (RNF213)* was the first MMD-associated gene reported, particularly in the Asian populations [8, 9]. A large-scale whole exome sequencing analysis of 125 ethnically diverse MMD patients revealed *ZXD family zinc finger C (ZXDC)* and *obscurin (OBSCN)* as highly enriched variants in the Caucasian MMD population and non-*RNF213* founder mutation cases [10]. Recently, another whole exome sequencing analysis of 5 familial MMD cases reported *titin* (*TTN*) as a new familial gene associated with MMD [11]. Studies have also indicated the involvement of circulating factors in the development of MMD [12–14]. Higher plasma concentrations of vascular endothelial growth factor, matrix metalloproteinase, hepatocyte growth factor, and interleukin-1β have been reported in MMD [12, 13, 15, 16]. Increased level of basic fibroblast growth factor has been reported in the cerebrospinal fluid of these patients [17] and has been demonstrated to predict response to surgery after indirect revascularization [18]. Cellular retinoic acid-binding protein (CRABP)-I has also been demonstrated to be elevated in the cerebrospinal fluid (CSF) of patients with MMD, making it a potential target in the pathogenesis of MMD [19].

Another study aimed to identify specific biomarkers in the CSF of patients with MMD using the quantitative proteome technique [20]. Two proteins were significantly upregulated, and 2 other proteins were downregulated in the CSF. Mass spectrometry analysis revealed that haptoglobin and α-1-B-glycoprotein were upregulated, indicating inflammation and/or angiogenesis in MMD. On the other hand, apolipoprotein-E (apoE), apoE precursor, and apolipoprotein-J (apoJ) were significantly downregulated in the CSF. The overexpression of haptoglobin was thought to indicate inflammation and/or angiogenesis in MMD. The downregulation of apoE and apoJ strongly suggested a critical role of lipid metabolism in the development and progression of MMD [20].

Two distinct subtypes of MMD have been described: ischemic and hemorrhagic [6, 7]. Significant clinical differences exist between the two subtypes, with fewer studies demonstrating differences at the molecular level. Understanding the mechanisms of MMD pathogenesis is an important step toward developing future nonsurgical approaches or drug-based treatments to arrest disease progression. In this study, we performed a comprehensive multiplex Luminex assay to investigate the molecular profile of 62 secreted proteins in the CSF of both subtypes of MMD patients (ischemic and hemorrhagic) and examined the relationship between the levels of these secreted factors with preoperative perfusion status, the extent of postoperative angiographic revascularization, and eventual functional outcomes.

## Materials and Methods

### Patient Population and Surgical Procedure

This study was approved by the Institutional Review Board of Stanford Medicine (IRB-12625), and patient consent was obtained. Patients were identified retrospectively from a prospectively maintained database. All patients had been surgically treated by the senior author (GKS). Intraoperative CSF was collected from the intracranial subarachnoid space (Table 1) from 32 controls (Chiari malformation and or cranial nerve hyperactive syndromes) and 73 MMD patients (ischemic group *n* = 37; hemorrhagic group *n* = 34) after opening the dura. MMD patients underwent combined direct and indirect STA-MCA (superficial temporal artery to middle cerebral artery) revascularization, using the direct STA donor also as an indirect graft by harvesting a long segment to lay on the cortical surface. Demographic information, including sex, age, and race, was documented (Table 1). Participant eligibility/criteria: CSF samples were selected from patients diagnosed with ischemic or hemorrhagic MMD who underwent surgical intervention. We used consecutive sampling of patients who were identified retrospectively. Sample size for each MMD group was determined by the fixed number of patients we had in the control group. MMD patients were presented with either ischemic or hemorrhagic MMD in line with their presenting symptomatology and imaging findings. We did not detail specific ischemic or hemorrhagic subtypes since in patients with ischemic MMD, there may be the presence of old infarcts/T2 MR white matter changes in combination with ongoing evidence of TIAs or new stroke. Hemorrhagic MMD patients often present with combinations of parenchymal, subarachnoid, and intraventricular hemorrhage.

**Table 1:** Clinical information and demographic characteristics of study participants. The table shows the mean age range, percentage of females, and racial breakdown in the study groups

	MMD-I *N* = 37	MMD-H *N* = 34	Controls *N* = 32
Mean age (range), *years*	40 (19 − 63)	40.7 (19 − 61)	43.6 (16 − 64)
Female, *n*	30 (76.9%)	22 (64.7%)	23 (71.9%)
Race			
Caucasian, *n*	20 (51.3%)	15 (41.1%)	10 (31.2%)
Others, *n*	17 (48.7%)	19 (55.9%)	13 (68.8%)

### Clinical and Radiological Data

Preoperatively, cerebral perfusion maps, including acetazolamide challenge (Xenon CT or MRI), were used to obtain information regarding the extent of preoperative cerebrovascular reserve [21, 22]. This was simplified into normal augmentation, no augmentation, or steal. Prior to surgical treatment, patient’s performance status was determined by the modified Rankin score (mRS; 0–5). After surgery, patients were clinically evaluated at 1 week postoperatively with further evaluations being carried out at 6 months, 3 years, and 10 years, including MRI, cerebral perfusion studies (Xenon CT and/or MR perfusion), and digital subtraction angiography (DSA). We used the 6-month DSA, including selective external carotid artery injections, to evaluate bypass patency and grade the extent of revascularization using Matsushima criteria (angiograde) (A: > 2/3rd of the MCA territory; B: between 1/3rd and 2/3rd, and C: < 1/3rd of the territory) [23]. In patients with bilateral revascularization, we selected the side with better revascularization to determine the Matsushima scale. In addition to the clinic follow-up, all patients were contacted via telephone to update their clinical and performance status.

### Luminex Human 62-plex Assay

Following intra-op collection, CSF samples were kept on ice and centrifuged for 15 min at 1500 rpm in a refrigerated centrifuge. Samples were then aliquoted into low protein binding tubes and stored at – 80 °C. The CSF samples were then transported to the Human Immune Monitoring Center at Stanford University, and the human 62-plex Luminex assay (eBiosciences/Affymetrix/ThermoFisher) was processed accordingly to the manufacturer’s recommendations with modifications as described below. Briefly: Beads were added to a 96-well plate and washed in a BioTek ELx405 washer. Samples were added to the plate containing the mixed antibody-linked beads and incubated at room temperature for 1 h, followed by overnight incubation at 4 °C with shaking. Cold and room-temperature incubation steps were performed on an orbital shaker at 500–600 rpm. Following the overnight incubation, plates were washed in a BioTek ELx405 washer, and then a biotinylated detection antibody was added for 75 min at room temperature with shaking. The plate was washed as above, and streptavidin-PE was added. After incubation for 30 min at room temperature, a wash was performed as above, and reading buffer was added to the wells. Each sample was measured in duplicate. Plates were read using a FlexMap 3D with a lower bound of 50 beads per sample per cytokine. Custom assay control beads by Radix BioSolutions were added to all wells. The presence of blood was evaluated by visual examination of all the wells at the initial step after the samples were added to the plate. The solution appears pink if there was blood in the sample. We observed a low presence of blood in some of the samples, with significant blood contamination observed in 2 out of 37 patients in the MMD-I group and 5 out of 34 patients in the MMD-H group.

### CRABP1 ELISA

CRABP1 was also measured using ELISA (MyBiosource.com, Cat#MBS2023566, San Diego, CA) according to the manufacturer’s instructions. Briefly, 70 μl from each CSF sample was diluted in the 140 μl of 1XPBS, which provides sufficient volume for duplicates. Each well contained 100 μl of the diluted sample. The microplates were pre-coated with an antibody specific to CRABP1. Standards or samples were added to the appropriate microplate wells with a biotin-conjugated antibody specific to CRABP1. Next, avidin conjugated to horseradish peroxidase (HRP) was added to each microplate well and incubated. After TMB substrate incubation, the enzyme–substrate reaction was terminated by the addition of sulfuric acid solution, and the color change was measured spectrophotometrically at a wavelength of 450 nm. The concentration of CRABP1 in the samples was determined by comparing the O.D. of the samples to the standard curve.

### Statistical Analysis

All statistical analyses were completed in R (version 4.0.3). A chi-square test of independence was performed to examine the relationship between post-surgery angiograde and various clinical characteristics (age, sex, side of disease, mRS initial, mRS final, race, and preop perfusion status). Luminex data were expressed as mean fluorescence intensity (MFI) and processed for quality check and log2 transformed. Analysis of secreted CSF protein levels from the Luminex assay was performed using a linear model adjusted for CHEX4 (nonspecific binding), age, and sex followed by least squares means (LSMeans) test with Tukey *p*-value adjustment method for multiple comparisons between control vs MMD-H, control vs MMD-I and ischemic vs hemorrhagic. An ordered probit model was used to ascertain the relationship between CSF protein level and the extent of revascularization (angiograde) as the main outcome. Independent variables in the logistic regression were sex, age, side of the disease, and CSF protein levels.

## Results

### Study Population

The study population (Table 1) included 32 controls (Chiari malformation and/or cranial nerve hyperactive syndromes), 34 MMD hemorrhagic patients, and 37 MMD ischemic patients. Ethnicity was divided into two categories: Caucasians and non-Caucasians (Table 1). Overall, the study was relatively balanced except for sex, with a greater proportion of females in all three groups.

### Relationship Between Clinical Variables in MMD Patients

**Fig. 1:**
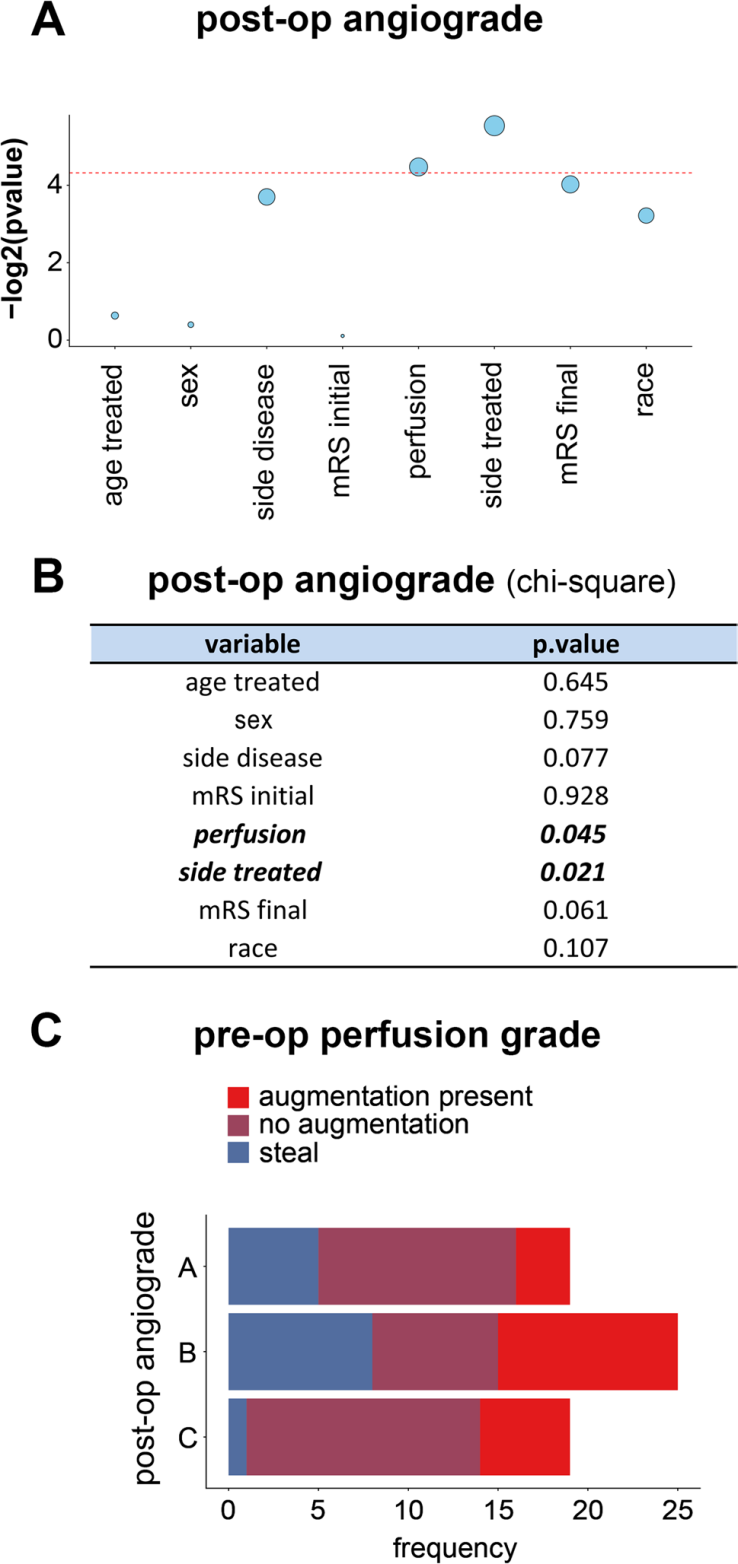
Relationship between angiograde and perfusion status in moyamoya patients. **A**, **B** The clinical variables examined included age at treatment, sex, side of disease, baseline perfusion status, side of treatment (unilateral versus bilateral), initial mRS, final mRS, and race. We found that postoperative angiograde was significantly associated with preoperative perfusion status and side of treatment. **C** Stack plot shows the frequency of perfusion grade in each angiograde score. Note that patients with poor perfusion status at baseline had better postoperative angiograde

The clinical variables we examined included age at treatment, sex, side of disease, baseline preop perfusion status, side of treatment (unilateral versus bilateral), initial mRS, final mRS, and race. A chi-square test of independence was performed to examine the relationship between the extent of revascularization (postop angiograde) and other clinical variables (Fig. 1A, B). Angiograde was significantly related to the side treated (the side of the brain that received surgical treatment) and preoperative perfusion status. Higher angiograde was observed in patients undergoing bilateral cerebral revascularization (*p* < 0.05). Further analysis revealed that patients with poor cerebral perfusion status preoperatively had higher postoperative angiograde (*p* < 0.05) (Fig. 1C). There was no significant relationship between final mRS and other clinical variables, with the exception of initial mRS (Supplementary Fig. 3).

### Secreted CSF Factors in Relation to MMD Subtypes

**Fig. 2:**
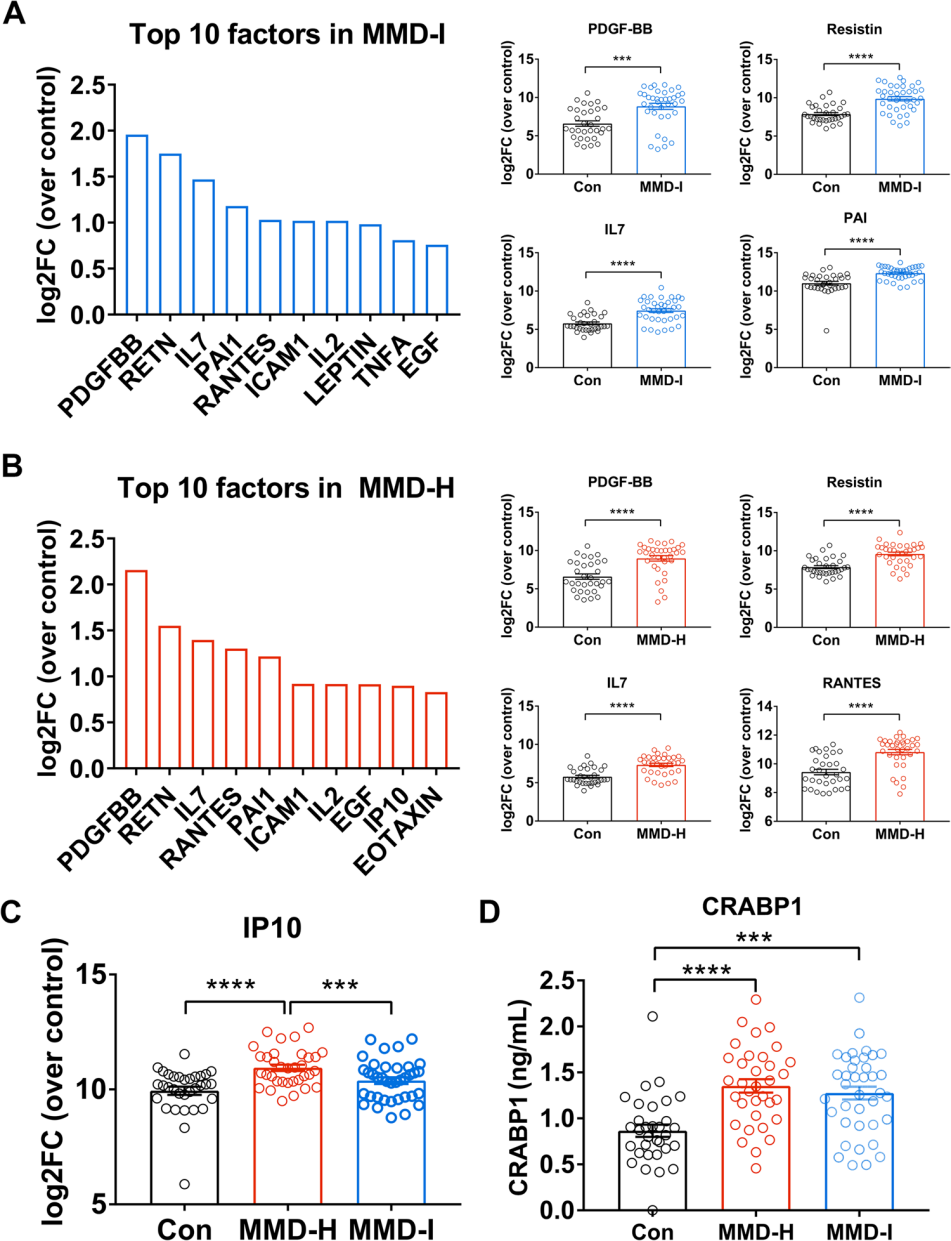
Top CSF secreted factors (**A**) in MMD ischemic (MMD-I) and MMD hemorrhagic (MMD-H) groups. A graph shows the top 10 factors in MMD-I and MMD-H (**B**) groups. Data are plotted as log2FC normalized over the control group. Bar graphs on the right depict the levels of top proteins (PDGF-BB, RETN, IL-7, and PAI-1) expressed as log2 MFI (mean fluorescence intensity). Data indicates the least square mean. Error bars are the 95% confidence of the LS mean. **p < 0.01, ***p < 0.001, and ****p < 0.0001, Tukey corrected. *N* = 32 for control, *n* = 37 for MMD-I, and *n* = 34 for MMD-H. **C** C-X-C motif chemokine 10 (CXCL10/IP10) was the only factor showing significant levels between MMD-I and MMD-H. **D** Bar graphs indicate mean protein levels of cellular retinoic acid binding protein 1 (CRABP1) in each group quantitated by ELISA. Dots represent the levels of CRABP1 in individual patients. ***p < 0.001 and ****p < 0.0001. One-way ANOVA followed by Bonferroni post hoc test

To identify key secreted factors in the CSF of MMD patients, we performed a comprehensive multiplex Luminex assay (62-plex) that could detect immune factors, cytokines, and growth factors. Luminex data (MFI) were plotted for each group for all factors to assess if they are normally distributed between the 3 groups (Supplementary Fig. 1). MFI for each factor were also plotted to assess if they are normally distributed (Supplementary Fig. 2). Forty-one secreted factors were significantly elevated in both MMD subtypes when compared to the non-MMD controls (Supplementary Table 1). Many of these secreted proteins have not been previously reported in MMD, including interleukins (IL-2, IL-4, IL-5, IL-7, IL-8, IL-9, IL-17, IL-18, IL-22, and IL-23) and C-X-C motif chemokines (CXCL1 and CXCL9) (Supplementary Table 1, *asterisks were added next to molecules that have not been previously reported in MMD). Among the top 10 secreted factors that were upregulated in the MMD-ischemic group were platelet-derived growth factor BB (PDGF-BB), resistin (RETN), interleukin-7 (IL-7), plasminogen activator inhibitor 1 (PAI-1), C–C motif chemokine ligand 5 (CCL5/ RANTES), intercellular adhesion molecule 1 (ICAM1), interleukin-2 (IL-2), leptin, tumor necrosis factor A (TNFA), and epidermal growth factor (EGF) (Fig. 2A). The top 10 secreted factors in the MMD-hemorrhagic group were very similar to MMD-ischemic group, including PDGF-BB, RETN, IL-7, CCL5/RANTES, PAI-1, ICAM1, IL-2, EGF, Chemokine C-X-C motif ligand 10 (CXCL10/IP10), and Eotaxin (Fig. 2B). The levels of these factors were normalized over control groups. When compared between MMD-I and MMD-H, the only molecule statistically significantly different between these two MMD subtypes is IP10 (Fig. 2C). See Supplementary Table 1 for details on *p*-value and log2 fold change for all three comparisons (con vs MMD-I, con vs MMD-H, and MMD-I vs MMD-H). Using ELISA, we also found a significant increase in the protein levels of cellular retinoic acid-binding protein (CRABP)-I (Fig. 2D), which is consistent with a previous report [19].

### Top Molecular and Cellular Functions in Relation to MMD Subtypes

**Fig. 3:**
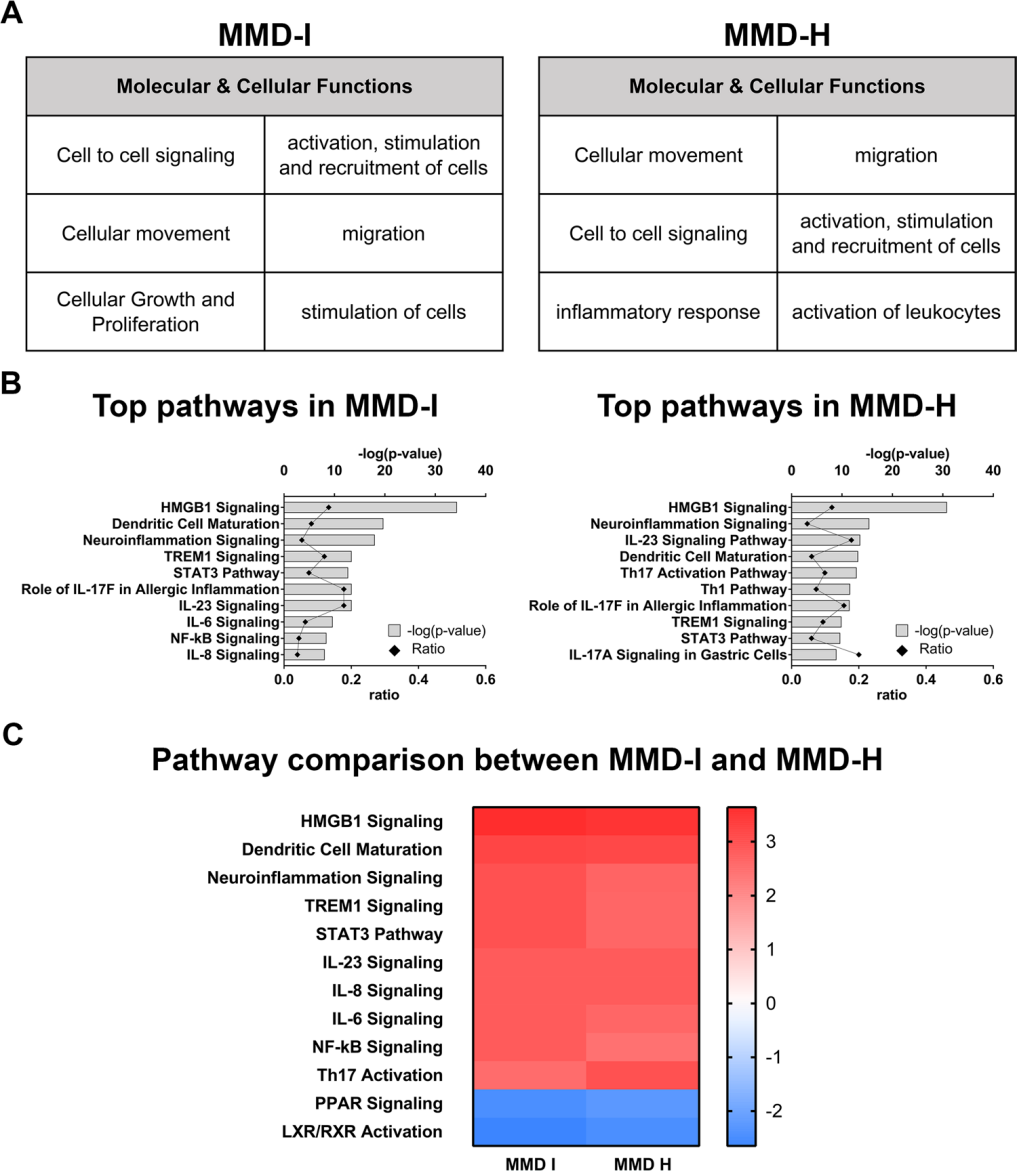
Top molecular functions and signaling pathways in MMD patients. **A** Table indicates the top molecular and cellular functions in the MMD-I and MMD-H groups. **B** Graphs show the top 10 canonical pathways from IPA® pathway analysis in MMD-I and MMD-H groups. *P*-values for the significance of each pathway were computed based on the number of molecules involved in the pathway by using Fisher’s exact test and negatively log10 transformed (represented in the upper *x*-axis). The ratio expresses the number of differentially expressed molecules over the total number of molecules in each canonical pathway based on the IPA knowledge database (represented in the lower *x*-axis). **C** Comparison analysis of common pathways between ischemic and hemorrhagic MMD. The color bar shows *z*-scores, where > 2 and < 2 indicate activation (red) and inhibition (blue) of each predicted pathway, respectively

To investigate the biological pathways involved in MMD, we further analyzed the molecular and cellular functions of these 41 secreted factors using the QIAGEN Ingenuity Pathway Analysis (IPA) (QIAGEN Inc., https://digitalinsights.qiagen.com/IPA) [24]. In the MMD ischemic group, the top molecular and cellular functions in ischemic MMD included cell-to-cell signaling, cellular movement, cellular growth and proliferation, activation, stimulation, and recruitment of cells and migration, and stimulation of cells (Fig. 3A). Similar molecular and cellular functions were observed in the hemorrhagic MMD, which also included inflammatory response and activation of leukocytes (Fig. 3A).

The top 10 canonical pathways as derived from the IPA pathway analysis were also similar between the ischemic and hemorrhagic subtypes of MMD (Fig. 3B). These pathways included high mobility group box 1 (HMGB1) signaling, neuroinflammation signaling, interleukin-23 (IL-23) signaling, dendritic cell maturation, T-helper 17 (Th17) signaling, and triggering receptor expressed on myeloid cells 1 (TREM1) signaling. Pathway analysis showed predicted activation in most of these pathways. Interestingly, two pathways were predicted to be inhibited: peroxisome proliferator-activated receptor (PPAR) signaling and liver X receptors (LXR)/retinoid X receptors (RXR) signaling (Fig. 3C). See Supplementary Table 2 for specific factors IPA categorized in each pathway.

### Secreted CSF Factors in Relation to Preoperative Perfusion Status and Postoperative Cerebral Revascularization

**Fig. 4:**
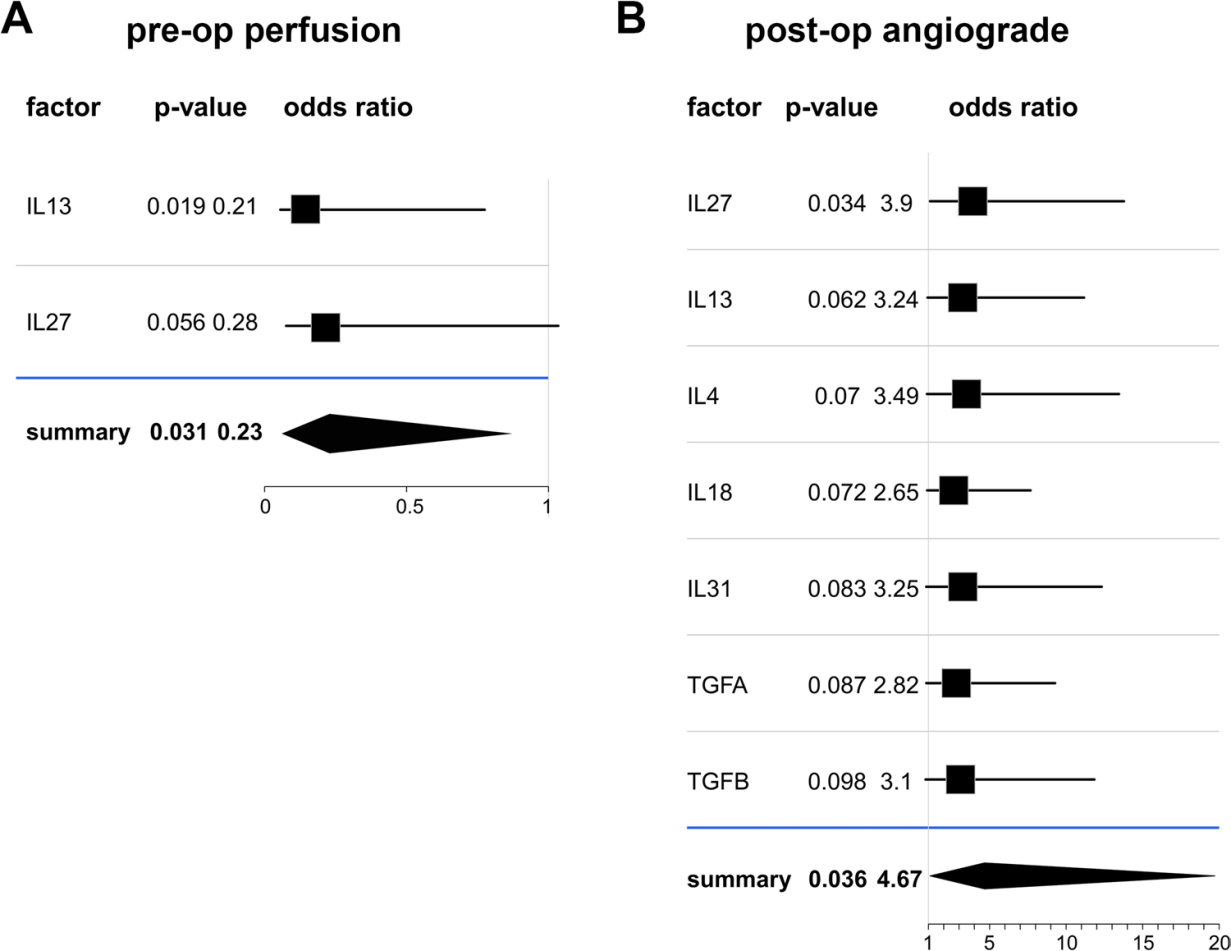
Relationship of secreted factors with preop perfusion status and postop angiograde. **A** Ordered probit regression displaying odds ratio assessing CSF factors in relation to preop perfusion status and postop degree of revascularization. A higher expression of IL-13 and IL-27 increases the odds of having worse preop perfusion status. **B** Higher expression of these 7 CSF factors increases the odds of having better postop angiograde outcome. Note that while an increase in these CSF factors increases the probability of better angiograde scores, it was the reverse for perfusion grade

Ordered probit regression analysis revealed that lower levels of IL-13 and IL-27 were associated with higher odds of better preoperative cerebral perfusion status (Fig. 4A) (overall OR = 0.23, *p*-value = 0.031). Greater postoperative revascularization as assessed by the Matsushima criteria was associated with increased levels of 7 secreted factors: interleukins (IL-27, IL-13, IL-4, IL-18, IL-31) and transforming growth factor alpha (TGFA) and beta (TGFB) (overall OR = 4.67, *p*-value = 0.036) (Fig. 4B). It was noteworthy that while an increase in these 7 CSF factors increased the probability of improved postoperative revascularization, the reverse was observed for the relationship between IL-13 and IL-27 and the preoperative cerebral perfusion status.

## Discussion

This is the first study to use a multiplex assay to comprehensively investigate 62 secreted factors in the CSF of two MMD subtypes (ischemic versus hemorrhagic). Our results revealed upregulation of forty-one secreted factors in both MMD subtypes when compared to non-MMD controls. Top molecules included PDGF-BB, RETN, IL-7, PAI-1, and CCL5/RANTES (Fig. 2A, B). Interestingly, our results revealed many novel proteins not previously reported in MMD, including interleukins (IL-2, IL-4, IL-5, IL-7, IL-8, IL-9, IL-17, IL-18, IL-22, and IL-23) and C-X-C motif chemokines (CXCL1 and CXCL9) (Supplementary Table 1). IP10 was the only factor that was significantly different between the two MMD subtypes (Fig. 2C). Bioinformatics analysis of these upregulated CSF factors demonstrated overlapping biofunctions in both MMD subtypes. Pathway analysis showed predicted activation in various signaling pathways involved in inflammation, immunity, and angiogenesis. Interestingly, two pathways showed predicted inhibition: peroxisome proliferator-activated receptor (PPAR) signaling and liver X receptors (LXR)/ retinoid X receptors (RXR) signaling (Fig. 3C). Orbit analysis revealed that IL-13 and IL-2 were negatively correlated with preoperative cerebral perfusion status, while 7 factors were positively correlated with the extent of postoperative revascularization (Fig. 4).

### Extent of Revascularization in Relation to Examined Clinical Variables

Analysis of the clinical variables in MMD patients showed a negative correlation between preoperative perfusion status and the extent of postoperative revascularization (Fig. 1). MMD patients with poor perfusion status at baseline had better postoperative revascularization. This may be related to the fact that patients with poor baseline cerebrovascular reserve have a greater need for improved blood flow and thus respond better to the revascularization procedure. In patients with bilateral revascularization, we selected the side with better revascularization to determine the Matsushima grade, potentially accounting for the higher angiograde in these patients. Ordered probit regression analysis demonstrated that higher levels of some of the CSF factors increased the overall odds of a better postoperative revascularization (Fig. 4).

### Pathophysiological Similarities Between the Ischemic and Hemorrhagic Subtypes of MMD

Despite the differences between the two clinical phenotypes of MMD, the two subtypes shared similar secreted factors and pathomechanisms. This was evident by the observation that the top secreted factors were similar in both groups, including CRABP-1, with overlapping biological functions and pathways. The only exception was IP-10, which was significantly higher in the hemorrhagic MMD subtype (Fig. 2C) compared to the ischemic MMD subtype. IP-10 is a cytokine involved in trafficking immune cells to inflammatory sites, and its pro-inflammatory role in multiple pathological conditions ranging from HIV and hepatitis to malignancy has been demonstrated [25]. IP10 also has an antiangiogenic effect through inhibition of endothelial cells [26–28]. Iron and heme have been reported to positively correlate with IP10 levels [29]. This is consistent with our observation of increased IP-10 in the MMD hemorrhagic group, which is likely due to the hemorrhagic events. We observed blood contamination in a higher number of CSF samples from the MMD hemorrhagic group, which could be related to the difference in IP10 between the two MMD subtypes.

Our study is unique in several ways. We performed a comprehensive investigation of 62 secreted proteins in the CSF of moyamoya disease patients, including immune factors, chemokines, cytokines, and growth factors. Our findings revealed many novel proteins not previously reported in MMD. We also used a non-MMD control group to compare to our MMD groups, which allowed us to identify MMD-specific factors. A non-MMD control group is more suitable than healthy control patients since any pathological disease states, as well as anesthesia, could alter immune-related and growth-related factors. Furthermore, we investigated two MMD subtypes, ischemic MMD and hemorrhagic MMD, in order to distinguish the factors different between these two subtypes. Importantly, we analyzed the relationship between these factors and preoperative cerebral perfusion status, as well as their relationship to the extent of postoperative revascularization. Some of the limitations in our study included retrospectively identified patients; sample size was determined from the limited number of control patients; non-MMD control patients had pathologies including Chiari or cranial nerve hyperactive syndromes, which may have affected the results; however, this is very unlikely; low blood contamination was observed in some of the CSF samples across all three groups, particularly in the MMD-hemorrhagic group. Thus, we could not exclude the effect of blood contamination on the one factor, IP-10, that was significantly different between the MMD-hemorrhagic and MMD-ischemic groups. A further limitation relates to the presence of comorbidities in our patient cohort, which included hypertension and other conditions like diabetes, and their relationship to either the MMD or neuroinflammatory cytokines. MMD is a chronic, lifelong disease, as are many of these comorbidities, with a potential interdependent relationship.

PDGF-BB was the top secreted factor observed in the CSF of MMD patients. This is supported by a previous study that observed increased PDGF-BB in the plasma of MMD patients [30]. PDGF-BB has been shown to be involved in regulating angiogenesis and endothelial growth [31, 32]. PDGF-BB has been demonstrated to stimulate cell migration and DNA synthesis in smooth muscle cells (SMCs) [33]. In the same study, interleukin-1 beta (IL-1 beta) significantly stimulated the migration and DNA synthesis of control SMCs, while it inhibited MMD SMC migration. The levels of IL-1 beta-induced nitric oxide production did not differ between moyamoya SMCs and control SMCs, suggesting that IL-1 beta inhibited the migration of moyamoya SMCs through a nitric oxide-independent pathway. The study concluded that the differential responses to PDGF and IL-1 in MMD SMCs are involved in the mechanism by which intimal thickening developed. Other implicated factors included Resistin with its pro-inflammatory effect and IL-7 with its influence on leukocyte development, malignancy, and viral infection [34, 35]. Consistent with a previous report, we also observed a significant increase of CRABP-1 in MMD groups. CRABP-1 has positive effect on smooth muscle cell proliferation and migration [19]. It has been speculated that these factors may contribute to the pathogenesis of MMD via inflammatory and immune-mediated pathways and may also have a role in the compensatory response leading to formation of collateral vessels and neovascularization as MMD progresses [36].

### Top Molecular and Cellular Functions in Relation to Disease Subtypes

Top molecular and cellular functions in ischemic MMD included cell-to-cell signaling; cellular movement; cellular growth and proliferation; activation, stimulation, and recruitment of cells; migration and stimulation of cells. In hemorrhagic MMD, the top molecular and cellular functions included were largely similar (Fig. 3). These functions, in addition to pointing to the underlying etiology of the disease process, likely reflect the compensatory response to progressive ICA obliteration in terms of neovascularization and collateralization. Despite different clinical presentations between the two subtypes, similar pathophysiological mechanisms reflect that a single nonsurgical therapeutic strategy may be beneficial in the future for both ischemic and hemorrhagic MMD, as is currently the case for surgical revascularization.

### CSF Factors in Relation to Preoperative Perfusion Status and Postoperative Extent of Cerebral Revascularization

We also identified molecular factors (Fig. 4) with direct clinical implications for MMD patients in terms of their relationship to preoperative cerebrovascular reserve (perfusion status) and postoperative extent of revascularization. Interestingly, higher expression of IL-13 and IL-27 predicted poor preoperative perfusion status and better postoperative extent of revascularization or angiograde. It was also independently observed that poor preoperative perfusion status led to better postoperative angiograde, consistent with the finding above. IL-13 has been noted to have inflammatory and anti-inflammatory properties [37]. IL-27 is another cytokine with diverse influences on immune responses [38]. Although it was initially linked with the development of Th1 responses, it is now recognized as a potent antagonist of different classes of inflammation through its ability to directly modify CD4( +) and CD8( +) T cell effector functions to induce IL-10. It has, therefore been implicated in inflammatory and anti-inflammatory pathways via the production of IL-10 [38]. Concomitant inflammatory and anti-inflammatory properties of these factors are consistent with the earlier observation that similar overlapping pathways contribute to both the pathogenesis and compensatory response in this progressive disease.

Our results revealed that a number of cytokines, chemokines, and growth factors are elevated in the CSF of MMD patients in both ischemic and hemorrhagic MMD cases. Many of these factors have not been previously reported in MMD. These secreted factors represent a complex interplay of underlying disease etiology as well as a compensatory response to the ischemic and hemorrhagic events, leading to formation of collateral vessels and neovascularization as the disease progresses. We found that some of the CSF factors are associated with preoperative perfusion status and the extent of postoperative revascularization. These findings implicate the involvement of immune/inflammatory-related pathways in the pathogenesis of MMD. Importantly, these highlighted factors could be potential future therapeutic targets and provide a basis for nonsurgical treatment of MMD.

### Supplementary Information

Below is the link to the electronic supplementary material.

Supplementary file1 (PDF 1942 KB)

## Data Availability

The datasets generated during and/or analysed during the current study are not publicly available due to patientprivacy but are available from the corresponding author on reasonable request.
