# Correction to: Comprehensive Profiling of Secreted Factors in the Cerebrospinal Fluid of Moyamoya Disease Patients

**DOI:** 10.1007/s12975-023-01149-1

**Published:** 2023-03-30

**Authors:** Kumar Abhinav, Alex G. Lee, Arjun V. Pendharkar, Mark Bigder, Anthony Bet, Yael Rosenberg-Hasson, Michelle Y. Cheng, Gary K. Steinberg

**Affiliations:** 1 Department of Neurosurgery, Stanford University School of Medicine, 1201 Welch Road, MSLS P305, Stanford, CA 94305 USA; 2 Stanford Stroke Center, Stanford University School of Medicine, Stanford, CA USA; 3 Present Address: Department of Neurosurgery, Bristol Institute of Clinical Neuroscience, Southmead Hospital, Bristol, UK; 4 Division of Hematology and Oncology, Department of Pediatrics, University of California, San Francisco, CA USA; 5 Human Immune Monitoring Center, Stanford University School of Medicine, Stanford, CA USA

**Correction to: Translational Stroke Research**

10.1007/s12975-023-01135-7

In the article “Comprehensive Profiling of Secreted Factors in the Cerebrospinal Fluid of Moyamoya Disease Patients” by Abhinav et al. 2023, there was an error in the second paragraph of the introduction. The percentage written in this sentence “Approximately 85–90% of MMD patients exhibit a familial occurrence” is incorrect. The correct sentence should be “Approximately 10–15% of MMD patients exhibit a familial occurrence.”

The original article has been corrected.

