# Supplementary material for: Comprehensive Profiling of Secreted Factors in the Cerebrospinal Fluid of Moyamoya Disease Patients
Source: Transl Stroke Res. 2023 Feb 6;15(2):399–408. doi: 10.1007/s12975-023-01135-7 (PMC10891229; doi:10.1007/s12975-023-01135-7)
Supplement: Supplementary file 1 — Supplementary file1 (PDF 1942 KB) [file 12975_2023_1135_MOESM1_ESM.pdf]

Supplementary Information

Article Title:

Comprehensive Profiling of Secreted Factors in the Cerebrospinal Fluid of Moyamoya Disease Patients

Journal Name:

Translational Stroke Research

Authors:

Kumar Abhinav, MD, Alex G. Lee, PhD, Arjun V. Pendharkar, MD, Mark Bigder, MD, Anthony Bet, BS, Yael Rosenberg-Hasson, PhD, Michelle Y. Cheng, PhD, Gary K. Steinberg, MD, PhD

Corresponding Author:

Gary K. Steinberg, MD, PhD

Department of Neurosurgery, Stanford University School of Medicine

1201 Welch Road, MSLS P305

Stanford, CA 94305, USA

Telephone: 650-725-5562

Email: gsteinberg@stanford.edu

		Control vs Ischemic MMD		Control vs Hemorrhagic MMD		Ischemic vs Hemorrhagic MMD	
Gene symbol	Gene full name	p-value	log FC	p-value	log FC	p-value	log FC
BDNF*	Brain derived neurotrophic factor	0.00126	0.44478	0.00059	0.44917	0.99939	-0.00439
CD40LG	CD40 ligand	0.00006	0.58210	0.00033	0.53591	0.95746	0.04619
EGF	Epidermal growth factor	0.00540	0.75873	0.00025	0.91656	0.79594	-0.15783
ENA78/CXCL5	Chemokine (C-X-C motif) ligand 5	0.01663	0.32930	0.02633	0.28766	0.92942	0.04164
EOTAXIN/CCL11	Chemokine (C-C motif) 11	0.00020	0.70358	0.00001	0.83122	0.77324	-0.12764
FGFB	Fibroblast Growth Factor 2 (Basic)	0.00379	0.58053	0.00037	0.58014	1.00000	0.00039
GCSF	Granulocyte colony-stimulating factor	0.00015	0.51420	0.00132	0.39789	0.62609	0.11631
GMCSF	Granulocyte-macrophage colony-stimulating factor	0.00041	0.41383	0.00388	0.24499	0.29119	0.16884
GROA/CXCL1*	Chemokine (C-X-C motif) ligand 1	0.01952	0.19177	0.02526	0.20865	0.97492	-0.01688
HGF	hepatocyte growth factor	0.00161	0.38587	0.00185	0.40426	0.97878	-0.01839
ICAM1	Intercellular adhesion molecule 1	0.00031	1.02050	0.00013	0.92019	0.92207	0.10030
IFNA	Interferon alpha	0.04108	0.42730	0.00045	0.58948	0.54440	-0.16218
IFNB	Interferon beta	0.00010	0.58202	0.00033	0.53173	0.94811	0.05029
IL12P40	Interleukin 12B, subunit P40	0.00001	0.64680	0.00021	0.50162	0.57744	0.14518
IL17A*	Interleukin 17A	0.00030	0.65105	0.00016	0.53694	0.77683	0.11411
IL17F*	Interleukin 17F	0.00002	0.72796	0.00004	0.61222	0.75638	0.11574
IL18*	interleukin 18	0.00073	0.33835	0.00593	0.26711	0.75870	0.07124
IL1A	Interleukin 1 alpha	0.04259	0.18081	0.00419	0.23050	0.78136	-0.04969
IL1RA*	Interleukin 1 Receptor Antagonist	0.00146	0.48268	0.00524	0.36003	0.65527	0.12265
IL2*	Interleukin 2	0.00005	1.01930	0.00001	0.91776	0.90470	0.10154
IL22*	Interleukin 22	0.00932	0.21146	0.01273	0.18521	0.93010	0.02625
IL23*	Interleukin 23	0.00073	0.32556	0.00183	0.33437	0.99503	-0.00880
IL4*	Interleukin 4	0.00963	0.21316	0.02877	0.16116	0.75739	0.05200
IL5*	Interleukin 5	0.00010	0.60572	0.00028	0.46088	0.61185	0.14484
IL6	Interleukin 6	0.00142	0.44103	0.00512	0.43917	0.99992	0.00186
IL7*	Interleukin 7	0.00004	1.46914	0.00002	1.39671	0.97342	0.07243
IL8*	Interleukin 8	0.00010	0.52871	0.00651	0.44481	0.86163	0.08390
IL9*	Interleukin 9	0.00155	0.23884	0.01603	0.21265	0.94203	0.02620
IP10/CXCL10 *	Chemokine (C-X-C motif) ligand 10	0.36996	1.23438	0.00021	1.86515	0.00645	-0.59550
LEP	Leptin	0.01901	1.97436	0.71167	1.21905	0.18000	0.69563
LIF*	LIF interleukin 6 family cytokine	0.00051	0.49444	0.00134	0.43622	0.89638	0.05823
MCP3/CCL7*	Chemokine (C-C motif) Ligand 7	0.00004	0.41503	0.00026	0.32737	0.61308	0.08766
MIG/CXCL9*	Chemokine (C-X-C motif) ligand 9	0.00005	0.46447	0.00001	0.47359	0.99615	-0.00912
PAI1/Serpine1	Plasminogen activator inhibitor 1	0.00027	1.18125	0.00013	1.21773	0.97999	-0.03648
PDGFBB	Platelet-derived growth factor BB	0.00189	1.95672	0.00018	2.15813	0.92573	-0.20141
RANTES/CCL5	Chemokine (C-C motif) 5	0.00260	1.02928	0.00003	1.30416	0.60510	-0.27487
RETN	Resistin	0.00001	1.75074	0.00001	1.55027	0.84581	0.20047
SDF1A (CXCL12)	Chemokine (C-X-C motif) ligand 12	0.00017	0.26115	0.00023	0.26474	0.99677	-0.00359
TNFA	Tumor necrosis factor A	0.00003	0.80846	0.00002	0.71347	0.84478	0.09500
TRAIL*	TNF-related apoptosis-inducing ligand	0.00105	0.31619	0.00087	0.29705	0.97392	0.01914
VCAM1	Vascular cell adhesion molecule 1	0.00528	0.63407	0.00126	0.70437	0.73324	-0.07030
VEGF	Vascular endothelial growth factor	0.00568	0.25619	0.00360	0.23705	0.96915	0.01914
VEGFD*	Vascular endothelial growth factor D	0.00290	0.56303	0.00427	0.46910	0.84010	0.09394

Supplementary Table 1. Secreted CSF factors in moyamoya disease patients. Table shows a total of 43 secreted factors that were significantly altered in MMD. Leptin was significantly increased in only MMD-I when compared to control. IP10 was the only factor significantly different between MMD-I and MMD-H. The other 41 secreted CSF factors were significantly increased in both ischemic MMD and hemorrhagic MMD, when compared to control group. P-value and log FC (log fold change) are shown. Asterisk (*) indicates proteins that were not previously reported in MMD.

Pathway	Molecules involved in the pathway
HMGB1 Signaling	CD40LG,CSF2,CXCL8,ICAM1,IL12B,IL15,IL17A,IL17F,IL18,IL1A,IL1B,IL1R1 IL2,IL4,IL5,IL6,LEP,LIF,SERPINE1,TNF,TNFSF10,VCAM1
Dendritic Cell Maturation	CD40LG,CSF2,ICAM1,IFNA1/IFNA13,IFNB1,IL10,IL12B,IL15,IL18,IL1A,IL1B IL23A,IL6,LEP,TNF
Neuroinflammation Signaling Pathway	BDNF,CCL5,CXCL12,CXCL8,ICAM1,IFNA1/IFNA13,IFNB1,IL10,IL12B,IL18 IL1B,IL1R1,IL4,IL6,TNF,VCAM1
TREM1 Signaling	CCL7,CSF2,CXCL8,ICAM1,IL10,IL18,IL1B,IL6,TNF
STAT3 Pathway	EGF,FGF2,HGF,IL1A,IL1B,IL1R1,IL9,PDGFB,TGFA,VEGFA
IL-23 Signaling Pathway	CSF2,IL12B,IL17A,IL17F,IL1B,IL22,IL23A,TNF
IL-8 Signaling	CXCL1,CXCL8,EGF,ICAM1,IL9,VCAM1,VEGFA,VEGFD
IL-6 Signaling	CXCL8,IL18,IL1A,IL1B,IL1R1,IL6,TNF,VEGFA
NF-κB Signaling	CD40LG,EGF,IL18,IL1A,IL1B,IL1R1,TGFA,TNF
Th17 Activation Pathway	CSF2,IL10,IL12B,IL17A,IL17F,IL1B,IL1R1,IL22,IL23A,IL6
PPAR Signaling	CCL7,IL18,IL1A,IL1B,IL1R1,IL6,TNF
LXR/RXR Activation	IL18,IL1A,IL1B,IL1R1,PDGFB,TNF

Supplementary Table 2. Top canonical pathways altered in both MMD subtypes. Table shows top pathways activated or inhibited by the upregulated CSF factors in MMD. Specific molecules in each pathway are listed (categorized by QIAGEN IPA software).

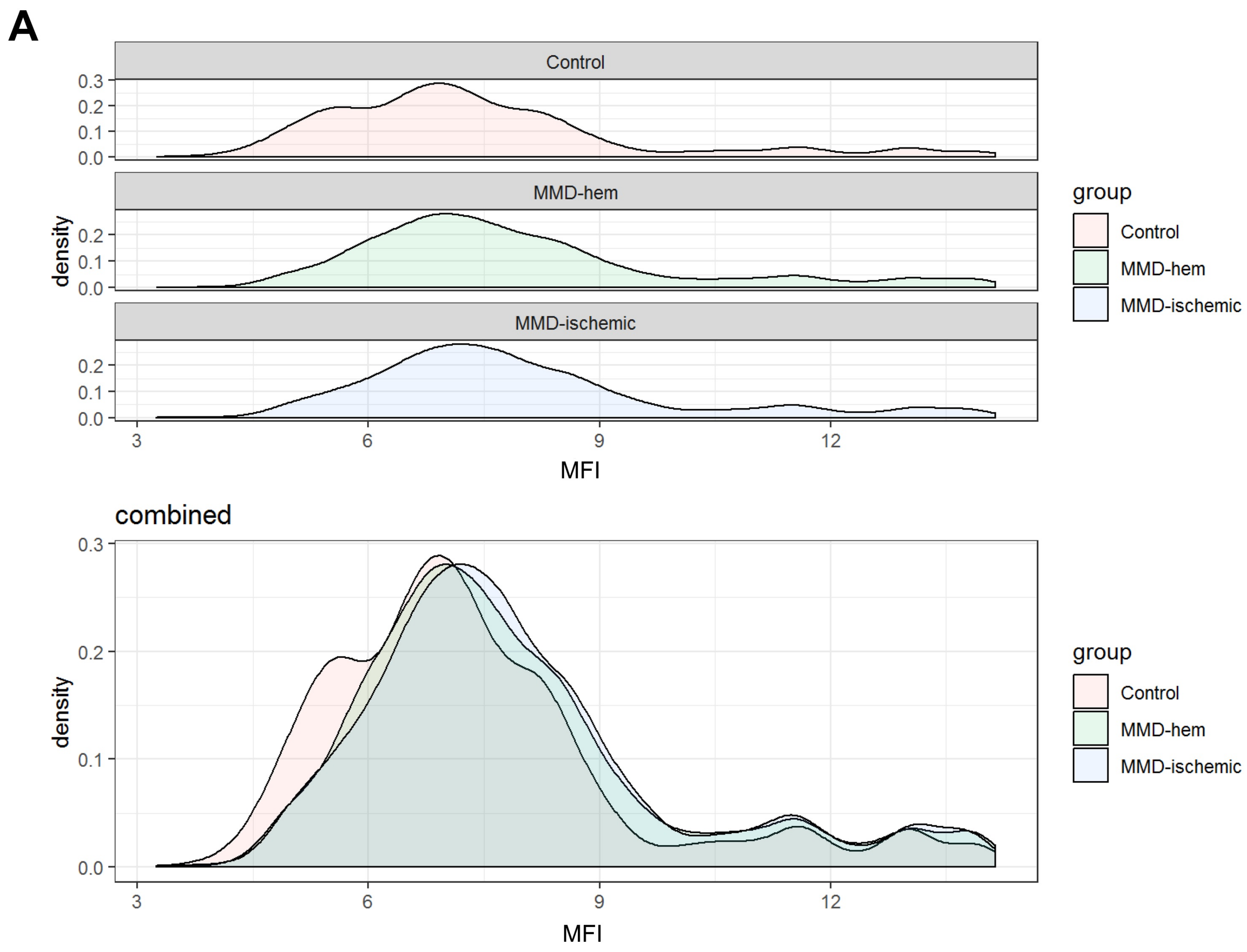

Supplementary Figure 1. Distribution density plot of secreted factors between groups. A, Density plot showing distribution of secreted factor levels (expressed as MFI) in control, MMD-hemorrhagic and MMD ischemic groups. B, Same data from all three groups plotted together.

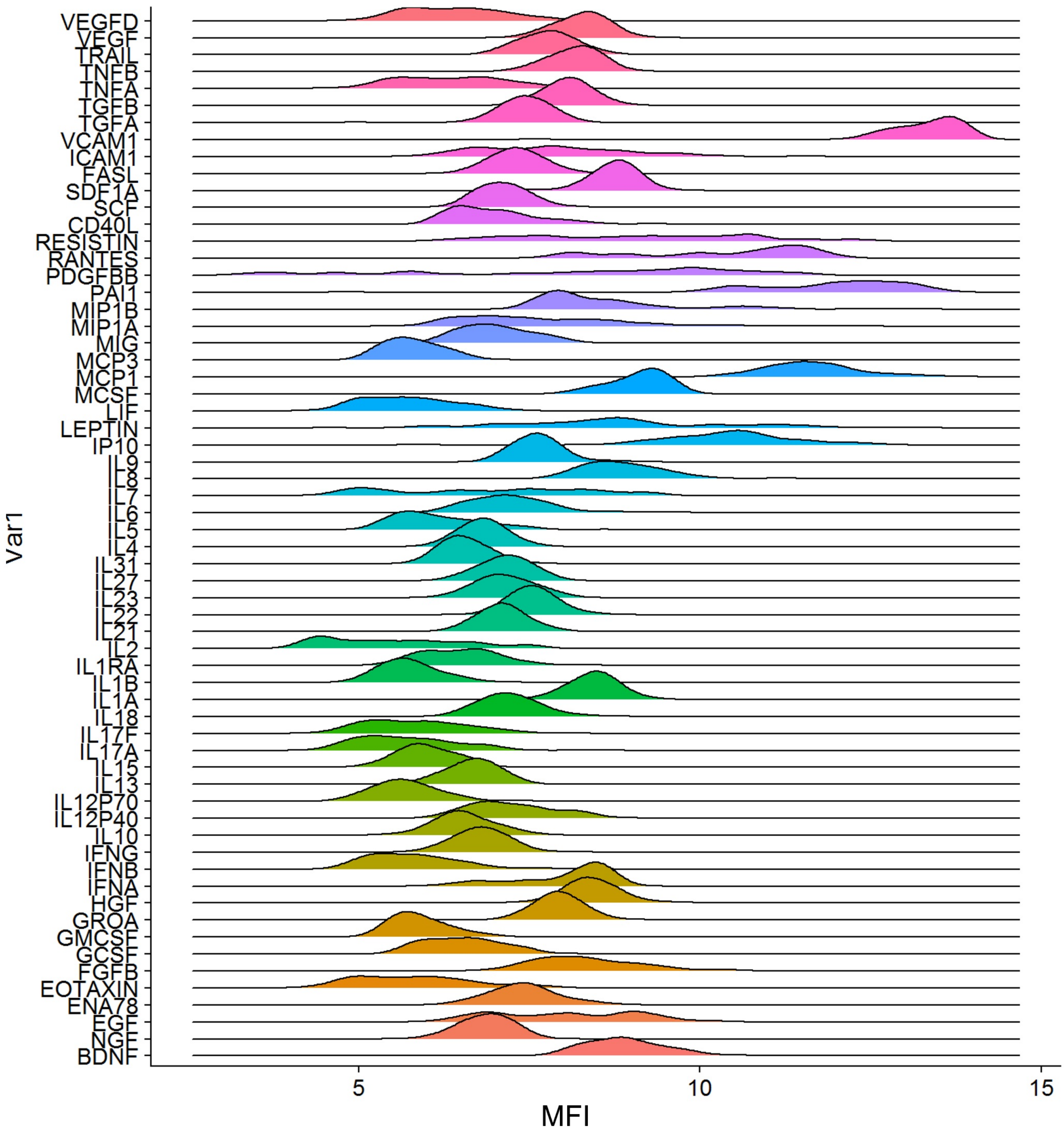

Supplementary Figure 2. Distribution density plot of individual secreted factor in all groups. Density plot showing distribution of each secreted factor levels (expressed as MFI) in all groups (control, MMD-hemorrhagic and MMD ischemic).

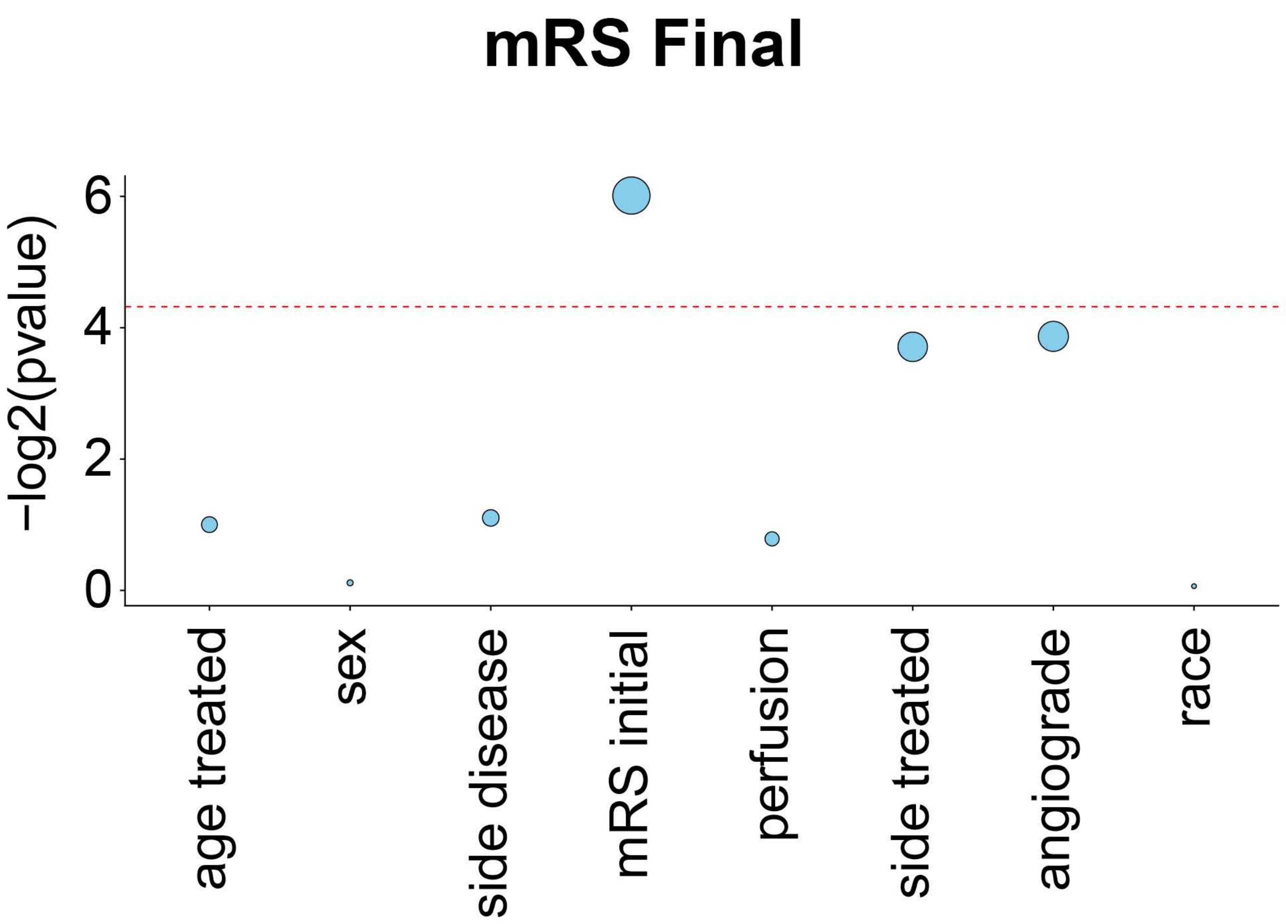

Supplementary Figure 3. Relationship of final mRS with other clinical variables. We found that final mRS has significant association with initial mRS, but not with other examined clinical variables.
